# Kaempferol induces autophagic cell death of hepatocellular carcinoma cells via activating AMPK signaling

**DOI:** 10.18632/oncotarget.21043

**Published:** 2017-09-16

**Authors:** Bing Han, Yi-Qun Yu, Qi-Lian Yang, Chun-Ying Shen, Xiao-Juan Wang

**Affiliations:** ^1^ Department of Pharmacy, Minhang Hospital, Fudan University, Shanghai, China

**Keywords:** kaempferol, hepatocellular carcinoma (HCC), AMPK, autophagy, MAGE6

## Abstract

In the present study, we demonstrate that Kaempferol inhibited survival and proliferation of established human hepatocellular carcinoma (HCC) cell lines (HepG2, Huh-7, BEL7402, and SMMC) and primary human HCC cells. Kaempferol treatment in HCC cells induced profound AMP-activated protein kinase (AMPK) activation, which led to Ulk1 phosphorylation, mTOR complex 1 inhibition and cell autophagy. Autophagy induction was reflected by Beclin-1/autophagy gene 5 upregulation and p62 degradation as well as light chain 3B (LC3B)-I to LC3B-II conversion and LC3B puncta formation. Inhibition of AMPK, via AMPKα1 shRNA or dominant negative mutation, reversed above signaling changes. AMPK inhibition also largely inhibited Kaempferol-induced cytotoxicity in HCC cells. Autophagy inhibition, by 3-methyaldenine or Beclin-1 shRNA, also protected HCC cells from Kaempferol. Kaempferol downregulated melanoma antigen 6, the AMPK ubiquitin ligase, causing AMPKα1 stabilization and accumulation. We conclude that Kaempferol inhibits human HCC cells via activating AMPK signaling.

## INTRODUCTION

Hepatocellular carcinoma (HCC) is a major cause of cancer-related moralities [1–3]. HCC’s five-year survival has been poor for those with advanced and/or metastatic cancers [1–3]. Yet, the incidence of this devastating disease has been rising [1–3]. The clinical treatment options for human HCC are very limited [4, 5]. Development of more efficient anti-hepatocellular carcinoma (HCC) agent is desperately needed [4–9].

Kaempferol is one key flavonol that is present in different fruits and vegetables [10, 11]. It is also a well-known Traditional Chinese Medicine showing different pharmacologic activities against atherosclerosis and hyperlipidemia [10, 11]. More importantly, Kaempferol has displayed anti-cancer activity against a number of cancer cells in experimental settings [10, 11]. Its potential activity against human HCC cells, and more importantly, the underlying mechanisms are not fully studied [10, 11].

AMP-activated protein kinase (AMPK) is the primary energy sensor in mammalian cells [12, 13]. Under stress conditions, activation of AMPK is responsible for maintaining homeostasis of cellular energy at both cellular and physiology levels [12–14]. Recent studies have proposed an anti-cancer function following AMPK activation [15–17]. At the molecule level, activation of AMPK could inhibit human cancer cells [16, 17] via regulating its downstream effectors, including activating p53 to induce cell cycle arrest and apoptosis [18–20]. AMPK activation may inhibit oncogenic mammalian target of rapamycin (mTOR) complex 1 (mTORC1) activation [21]. Further, AMPK serves as a major upstream signaling for cell autophagy induction [22, 23]. Additionally, sustained AMPK activation shall cause degradation of several oncogenic proteins [24]. In the current study, we provide evidences to suggest that activation of AMPK is responsible for Kaempferol-induced anti-HCC cell activity *in vitro*.

## RESULTS

### Kaempferol inhibits survival of established and primary human HCC cells

**Figure 1:**
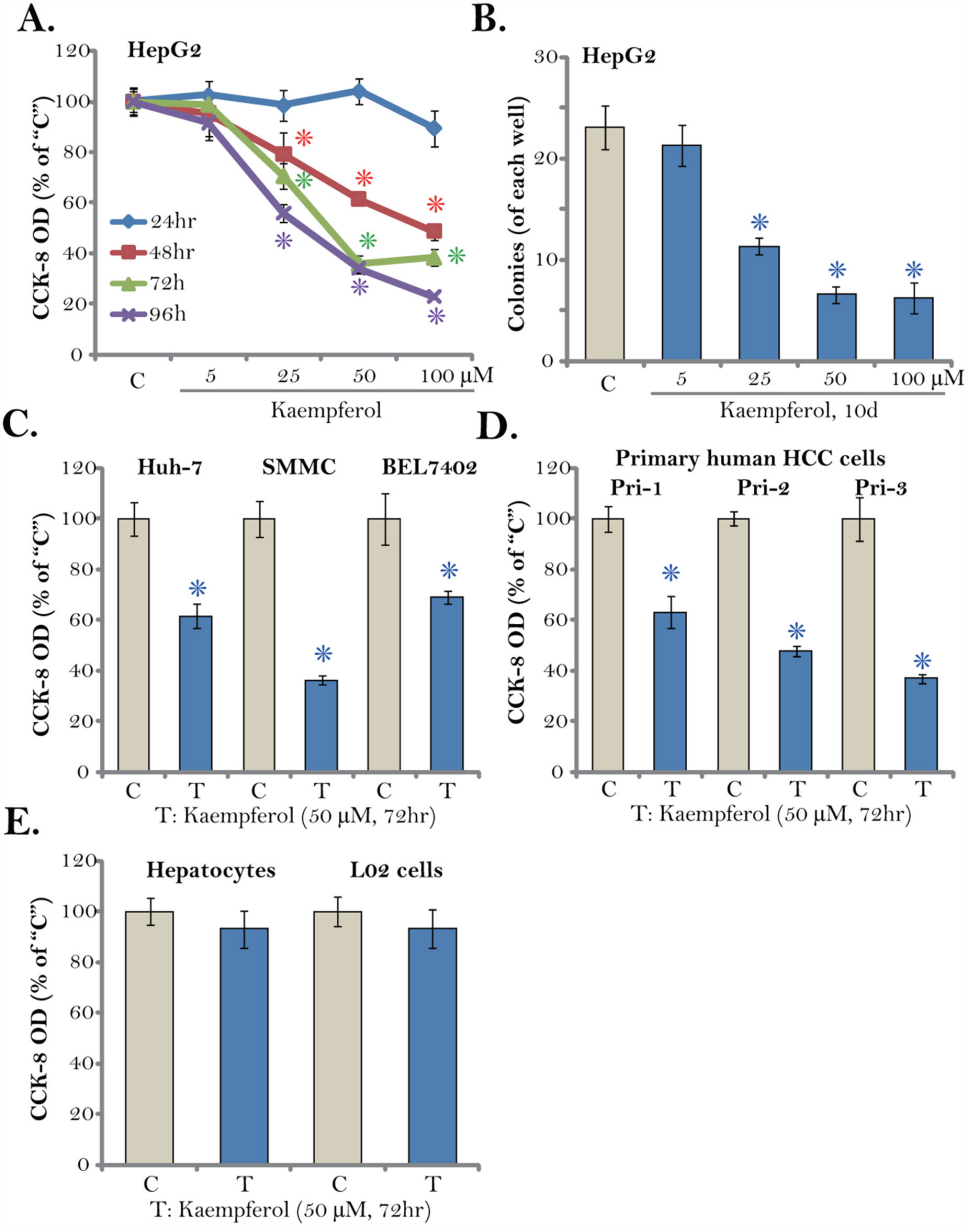
Kaempferol inhibits HCC cell survival Established human HCC cell lines (HepG2, Huh-7, BEL7402, and SMMC), the primary human HCC cells (“Pri-1/-2/-3”), as well as L02 hepatocytes (“L02”) and the primary human hepatocytes (“Hepatocytes”) were cultured in Kaempferol (5-100 μM)-containing medium for the indicated time. Cell survival was tested by CCK-8 assay **(A, C-E)** and colony formation assay (**B**, HepG2 cells). The data were presented as mean ± standard deviation (SD) (same for all figures). For each assay, n=5. “C” stands for untreated control group (Same for all figures). * *p* < 0.05 vs. “C” group. Experiments in this figure were repeated four times, and similar results were obtained.

First, Kaempferol, at different concentrations, was added to cultured HepG2 cells, which are well-established human HCC cells. Cells were then cultured in Kaempferol-containing medium for different time points (24-96 hours). Cell Counting Kit-8 (CCK-8) assay was performed to test cell survival. As displayed in Figure 1A, Kaempferol inhibited survival of HepG2 cells, and the CCK-8 OD was significantly decreased. Notably, the anti-survival activity by Kaempferol was both concentration- and time-dependent (Figure 1A). A low concentration of Kaempferol (5 μM) was in-effective against HepG2 cells (Figure 1A). The IC-50 of Kaempferol was close to 25-50 μM (Figure 1A). It would require at least 48 hours for Kaempferol (> 25 μM) to exert significant anti-survival effect (Figure 1A). Additionally, the number of HepG2 colonies was decreased sharply following Kaempferol (25-100 μM) treatment (Figure 1B), further confirming its anti-survival activity.

We also tested the potential activity of Kaempferol in other HCC cells. Three established human HCC cell lines, including Huh-7, BEL7402, and SMMC, were treated with Kaempferol (50 μM, for 72 hours). As shown in Figure 1C, cell survival, tested again by the CCK-8 OD, was significantly decreased after Kaempferol treatment. Next, a total of three lines of primary human HCC cells (gifts from Dr. Sun [25]) were cultured. These primary cancer cells were treated with/out Kaempferol (50 μM). CCK-8 assay results in Figure 1D confirmed that Kaempferol was anti-survival when added to all three lines of primary human HCC cells. On the other hand, very same Kaempferol (50 μM, 72 hours) treatment was yet non-cytotoxic to the L02 hepatocytes and primary human hepatocytes (provided by Dr. Fan [26]) (Figure 1E). The CCK-8 OD was almost unchanged following Kaempferol treatment in the hepatocytes (Figure 1E). These results demonstrate that Kaempferol inhibits survival of established and primary human HCC cells.

### Kaempferol inhibits HCC cell proliferation

**Figure 2:**
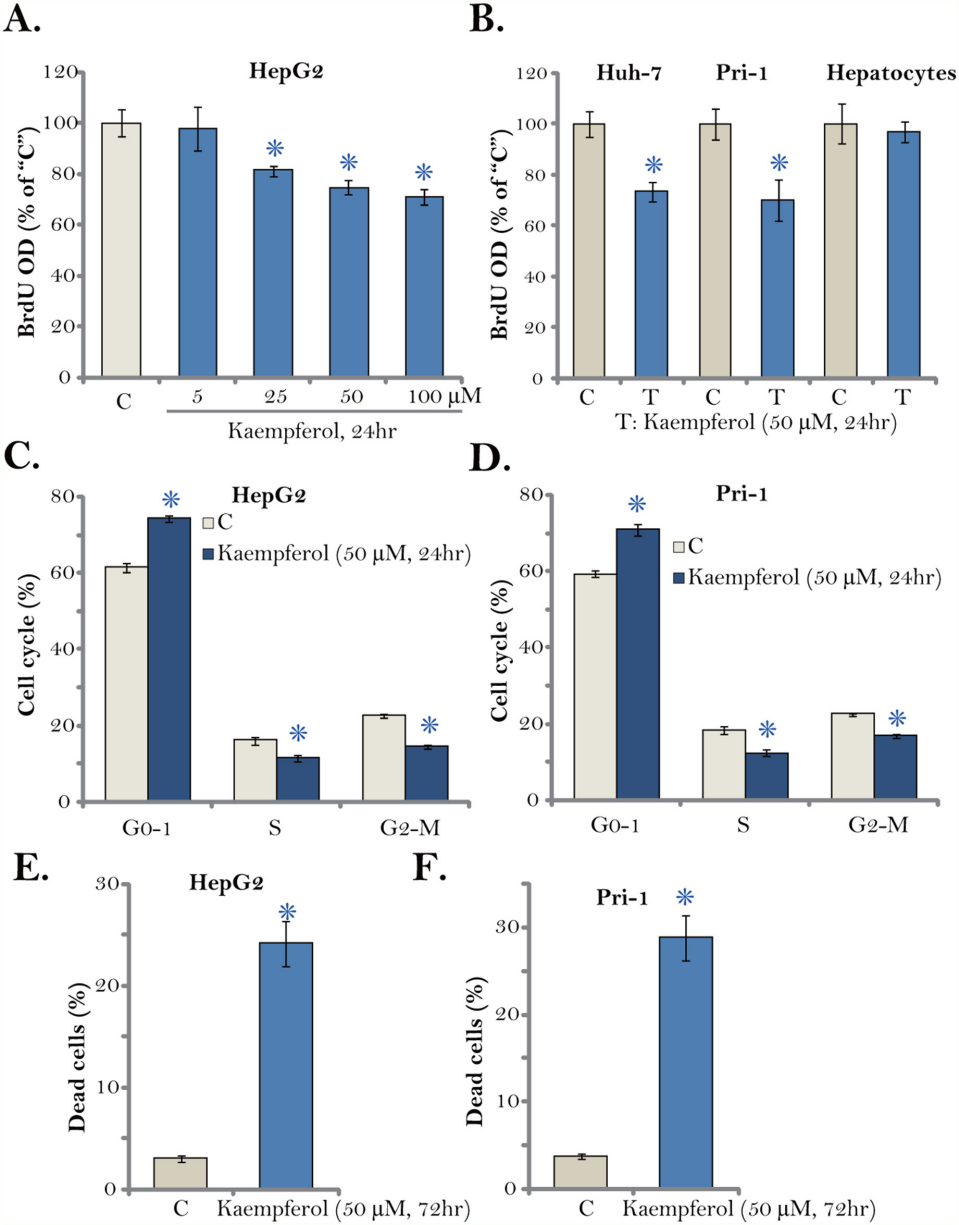
Kaempferol inhibits HCC cell proliferation Established human HCC cell lines (HepG2 and Huh-7), the primary human HCC cells (“Pri-1”), or the primary human hepatocytes (“Hepatocytes”) were cultured in Kaempferol (5-100 μM)-containing medium for the indicated time. Cell proliferation (BrdU ELISA assay, **A**-**B**), cell cycle distribution (FACS assay, **C** and **D)** and cell death (Trypan blue staining assay, **E** and **F)** were tested. For each assay, n=5. * *p* < 0.05 vs. “C” group. Experiments in this figure were repeated three times, and similar results were obtained.

The Kaempferol-induced effect on HCC cell proliferation was tested next. 5-bromo-2’-deoxyuridine (BrdU) incorporation is a well-established marker of cell proliferation. As displayed in Figure 2A, treatment with Kaempferol dose-dependently decreased BrdU ELISA OD in HepG2 cells. Proliferation inhibition was significant at 24 hours after Kaempferol (25-100 μM) treatment, when no significant cytotoxicity was noticed (Figure 1A). Similarly, Kaempferol (50 μM) was also anti-proliferative when added to Huh-7 cells and primary human HCC cells (“Pri-1”), as BrdU ELISA OD was decreased (Figure 2B). Further, cell cycle distribution experimental results showed that after Kaempferol treatment, the percentages of S and G2-M phase HepG2 cells were decreased, and G1 phase cell percentage was increased, suggesting G1-S cell cycle arrest (Figure 2C). The very similar G1-S arrest effect by Kaempferol was also observed in the primary HCC cells (“Pri-1”, Figure 2D). It should be noted that Kaempferol (50 μM) treatment induced HepG2 and primary human HCC (“Pri-1”) cell death (Figure 2E and 2F), the latter was reflected by the trypan blue staining assay.

### Kaempferol fails to induce HCC cell apoptosis

**Figure 3:**
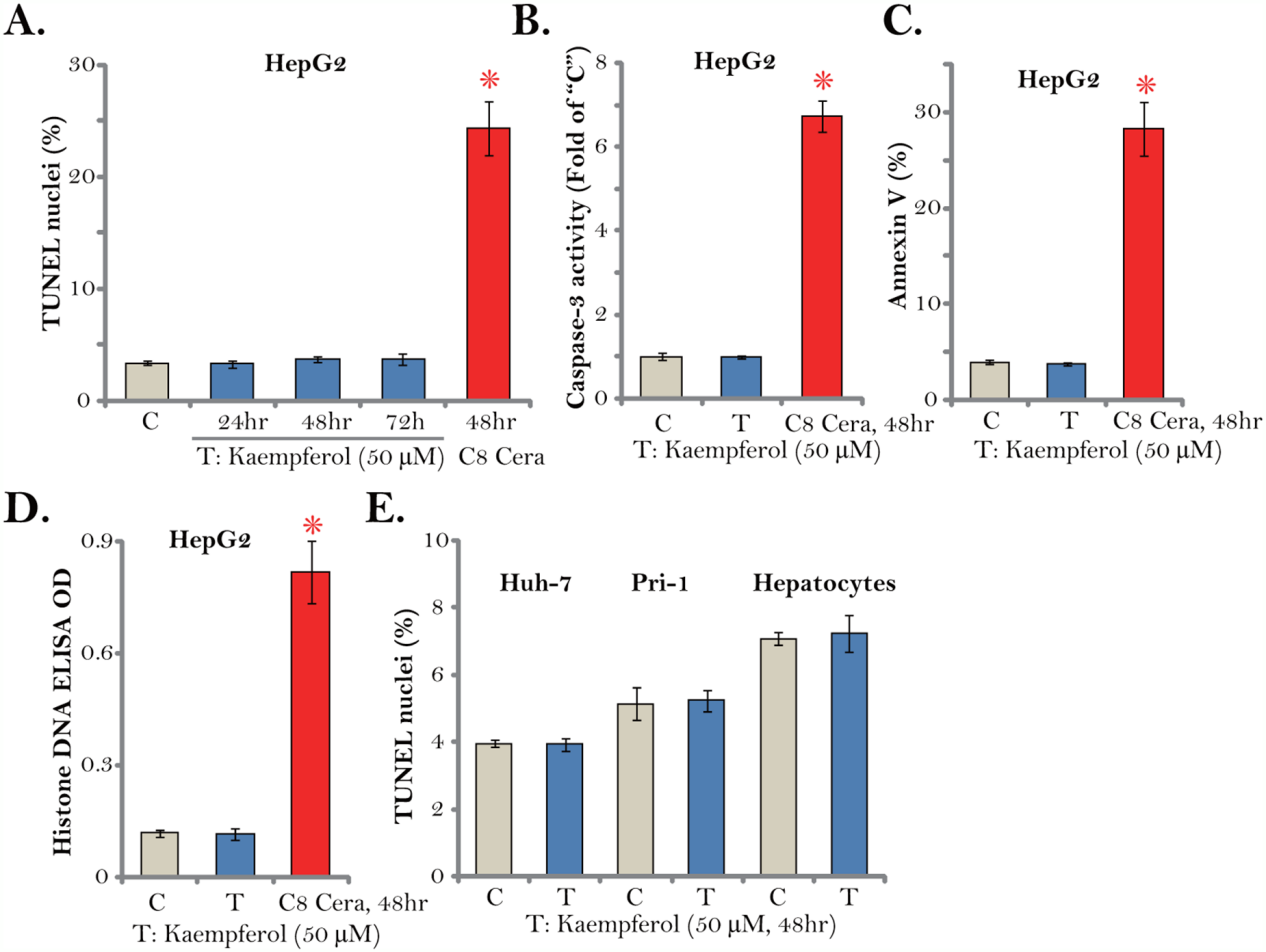
Kaempferol fails to induce HCC cell apoptosis Established human HCC cell lines (HepG2 and Huh-7), the primary human HCC cells (“Pri-1”), or the primary human hepatocytes (“Hepatocytes”) were cultured in Kaempferol (50 μM)- or C8 ceramide (“C8 Cera”, 25 μM)-containing medium for the indicated time. Cell apoptosis was tested by the assays mentioned in the text. For each assay, n=5. * *p* < 0.05 vs. “C” group. Experiments in this figure were repeated four times, and similar results were obtained.

Cell apoptosis activation could be an important cause of cell death and proliferation inhibition. We therefore tested apoptosis in Kaempferol-treated HCC cells. A set of various apoptosis assays were applied. The TUNEL assay results demonstrated that treatment with the cytotoxic Kaempferol (50 μM) for different time points (24/48/72 hours) failed to induce significant apoptosis activation in HepG2 cells (Figure 3A). Meanwhile, the caspase-3 activity (Figure 3B), the Annexin V ratio (Figure 3C) and the histone DNA ELISA OD (Figure 3D) were unchanged after Kaempferol treatment in HepG2 cells. These results imply that Kaempferol failed to induce significant apoptosis in HepG2 cells. On the other hand, C8 ceramide (25 μM, 48 hours), which was utilized as a positive control [27], induced profound apoptosis activation in HepG2 cells (Figure 3A-3D). Notably, Kaempferol treatment (50 μM, 48 hours) also failed to increase TUNEL nuclei ratio in Huh-7 cells and primary human HCC cells (“Pri-1”) (Figure 3E). Certainly no apoptosis was induced in Kaempferol-treated primary human hepatocytes (Figure 3E). These results suggest that Kaempferol fails to induce HCC cell apoptosis.

### Kaempferol induces autophagy activation in HCC cells

**Figure 4:**
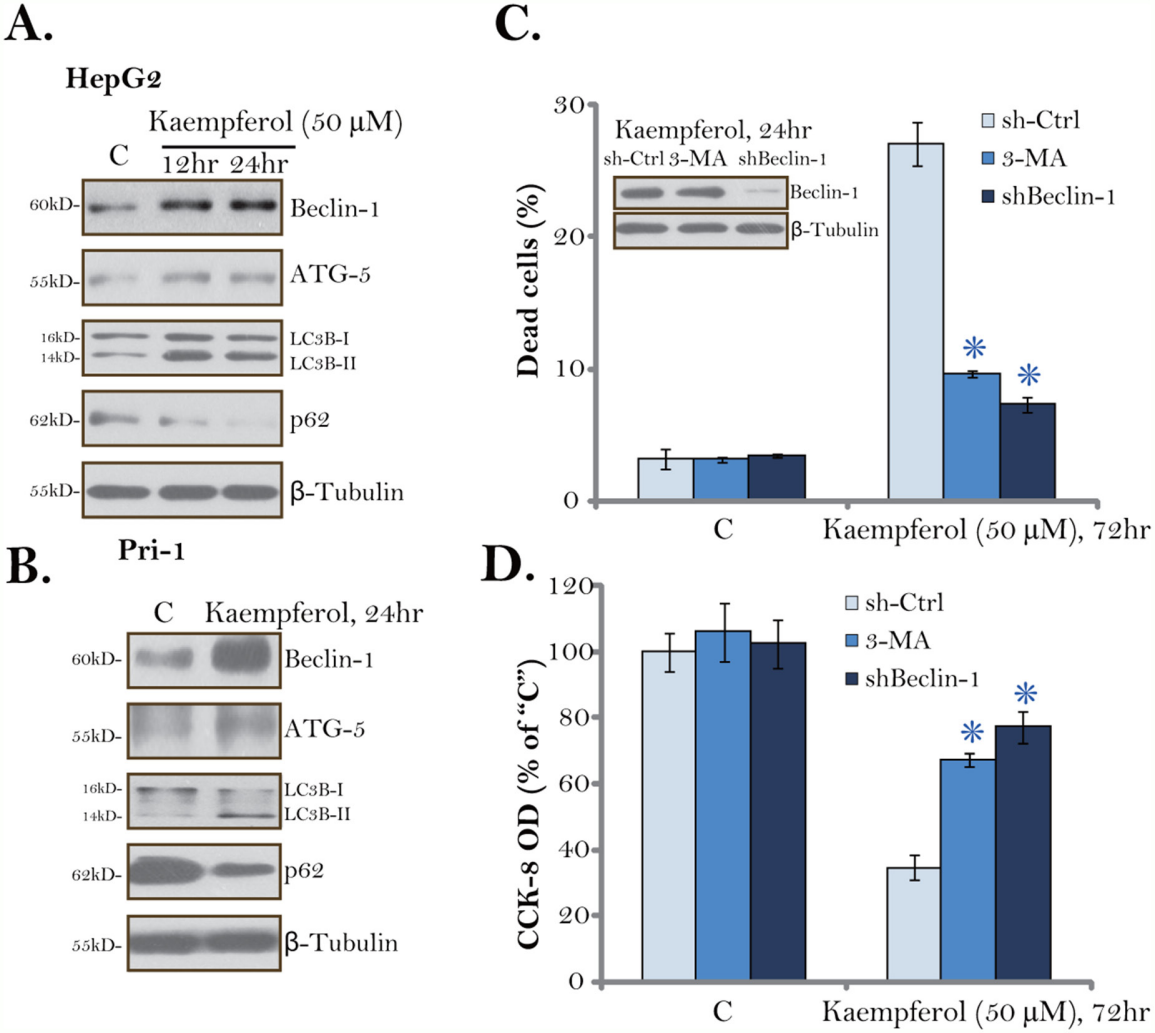
Kaempferol induces autophagy activation in HCC cells HepG2 cells **(A)** or primary human HCC cells (“Pri-1”, **B**) were cultured in Kaempferol (50 μM)-containing medium for the indicated time. Expressions of listed autophagy-associated proteins were shown. HepG2 cells were pre-treated with 3-methyladenine (3-MA, 5 mM, for 1 hour) or infected with Beclin-1 shRNA lentivirus, followed by Kaempferol (50 μM) treatment for additional 72 hours, cell death and cell viability were tested by Trypan blue staining assay (**C**, lower panel) and CCK-8 assay **(D)**, respectively. Expressions of Beclin-1 and β-Tubulin were also shown (C, upper panel). For each assay, n=5. “sh-Ctrl” stands for scramble control shRNA. * *p* < 0.05 vs. “sh-Ctrl” group. Experiments in this figure were repeated three times, and similar results were obtained.

Although autophagy could be pro-survival under certain circumstances, sustained autophagy activation shall induce cell death (autophagic cell death) [28–31]. The results mentioned above suggested that Kaempferol induced non-apoptotic cell death in HCC cells. We therefore tested potential autophagy induction in Kaempferol-treated HCC cells. Western blotting assay results showed that Kaempferol (50 μM) treatment induced Beclin-1/autophagy gene 5 (ATG-5) upregulation, light chain 3B (LC3B) LC3B-I to LC3B-II conversion as well as p62 degradation in HepG2 cells. The effect by Kaempferol was time-dependent, suggesting autophagy flux [32, 33]. The very similar changes on autophagy-associated proteins were also noticed in Kaempferol-treated primary HCC cells (Figure 4B). These results imply that Kaempferol induced autophagy activation in HCC cells.

To study the link between autophagy activation and Kaempferol-induced cytotoxicity, both pharmacological and genetic strategies were applied to inhibit autophagy. 3-methyladenine (3-MA) is a well-established autophagy inhibitor [34, 35]. Further, a number of studies utilized Beclin-1 shRNA to genetically block autophagy [35]. In the current study, we show that 3-MA pre-treatment (for 1 hour) or Beclin-1 shRNA largely inhibited Kaempferol-induced HepG2 cell death (Figure 4C) and viability reduction (Figure 4D). These results suggest that autophagy activation is required for Kaempferol-induced cytotoxicity in HCC cells. Notably, Beclin-1 shRNA indeed efficiently downregulated Beclin-1 in Kaempferol-treated HepG2 cells (Figure 4D). Treatment with 3-MA or Beclin-1 shRNA alone failed to change survival/death of HCC cells (Figure 4C and 4D).

### AMPK activation is required for Kaempferol-induced autophagy and cytotoxicity in HCC cells

**Figure 5:**
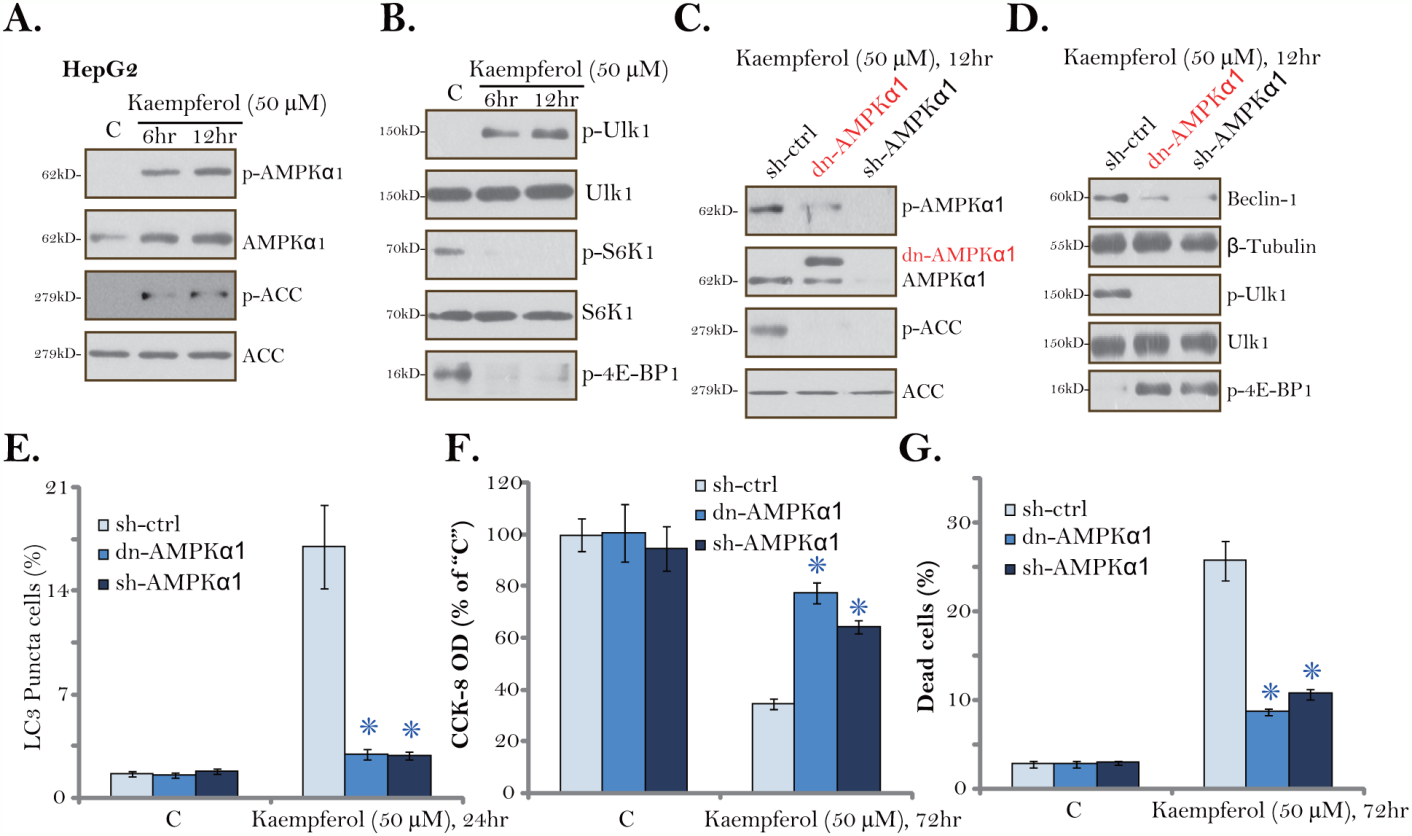
AMPK activation is required for Kaempferol-induced autophagy and cytotoxicity in HCC cells HepG2 cells were cultured in Kaempferol (50 μM)-containing medium for the indicated time. Expressions of listed proteins were shown **(A** and **B)**. Stable HepG2 cells, expressing AMPKα1 shRNA (“sh-AMPKα1”) or the dominant negative AMPKα1 (T172A, Flag-tagged, “dn-AMPKα1”), were cultured in Kaempferol (50 μM)-containing medium for the indicated time. Expressions of listed proteins were shown **(C** and **D)**. LC3 Puncta formation **(E)**, cell viability (CCK-8 assay, **F**) and cell death (Trypan blue staining assay, **G**) were also tested. For each assay, n=5. “sh-Ctrl” stands for scramble control shRNA. * *p* < 0.05 vs. “sh-Ctrl” group. Experiments in this figure were repeated three times, and similar results were obtained.

One possible upstream kinase to trigger autophagy is AMPK [22, 36, 37]. In the current study, we show that Kaempferol (50 μM) treatment in HepG2 cells induced significant AMPK activation (Figure 5A), which was reflected by phosphorylations (“p-”) of AMPKα1 (Thr-172) and its major downstream effector protein acetyl-CoA carboxylase (ACC, Ser-79) [38, 39]. Expression level of total AMPKα1, intriguingly, was also significantly increased by Kaempferol (Figure 5A), which might contribute to AMPK activation. AMPK is known to directly phosphorylate Ulk1 at Ser-317 to initiate autophagy [22, 23]. Here, significant Ulk1 phosphorylation (at Ser-317) was noticed in Kaempferol-treated HepG2 cells (Figure 5B). AMPK-mediated autophagy activation could also be due to mTORC1 inhibition [22, 40]. We show that, following Kaempferol treatment, the phosphorylations of two major mTORC1 substrates, S6K1 (S6 Kinase 1) and 4EBP1 (eIF4E-binding protein 1), were both significantly reduced (Figure 5B), confirming mTORC1 in-activation [41].

To study the link between AMPK activation and Kaempferol-induced actions in HCC cells, AMPKα1 shRNA and the dominant negative mutant AMPKα1 (dn-AMPKα1, T172A) [42] were applied to block AMPK activation. The lentiviral AMPKα1 shRNA or the dn-AMPKα1 (Flag-tagged, Figure 5C) was introduced to the HepG2 cells, and stable cells were established via puromycin selection. As displayed, Kaempferol-induced AMPK activation, or AMPKα1/ACC phosphorylations, were almost completely blocked by AMPKα1 shRNA or the dn-AMPKα1 (Figure 5C). More importantly, Kaempferol-induced Beclin-1 expression, Ulk1 phosphorylation and mTORC1 (“p-4EBP1”) inhibition were largely attenuated by AMPKα1 shRNA or dn-AMPKα1 (Figure 5D). Consequently, Kaempferol-induced autophagy induction, tested by the formation of LC3B-GFP puncta, was largely inhibited (Figure 5E). These results suggest that AMPK activation mediates Kaempferol-induced Ulk1 phosphorylation, mTORC1 inhibition and autophagy induction in HCC cells.

We next hypothesized that AMPK activation should also be important for Kaempferol-mediated cytotoxicity. Indeed, in the stable HepG2 cells with AMPKα1 shRNA or the dn-AMPKα1, Kaempferol-induced cell viability reduction (Figure 5F) and cell death (Figure 5G) were largely attenuated. Thus, AMPK in-activation or knockdown protected HepG2 cells from Kaempferol, confirming that AMPK activation is required for Kaempferol-induced cytotoxicity against HepG2 cells. AMPKα1 shRNA or dn-AMPKα1 alone failed to change HepG2 cell survival/death (Figure 5F and 5G).

### Kaempferol downregulates melanoma antigen6 (MAGE6) in HCC cells

**Figure 6:**
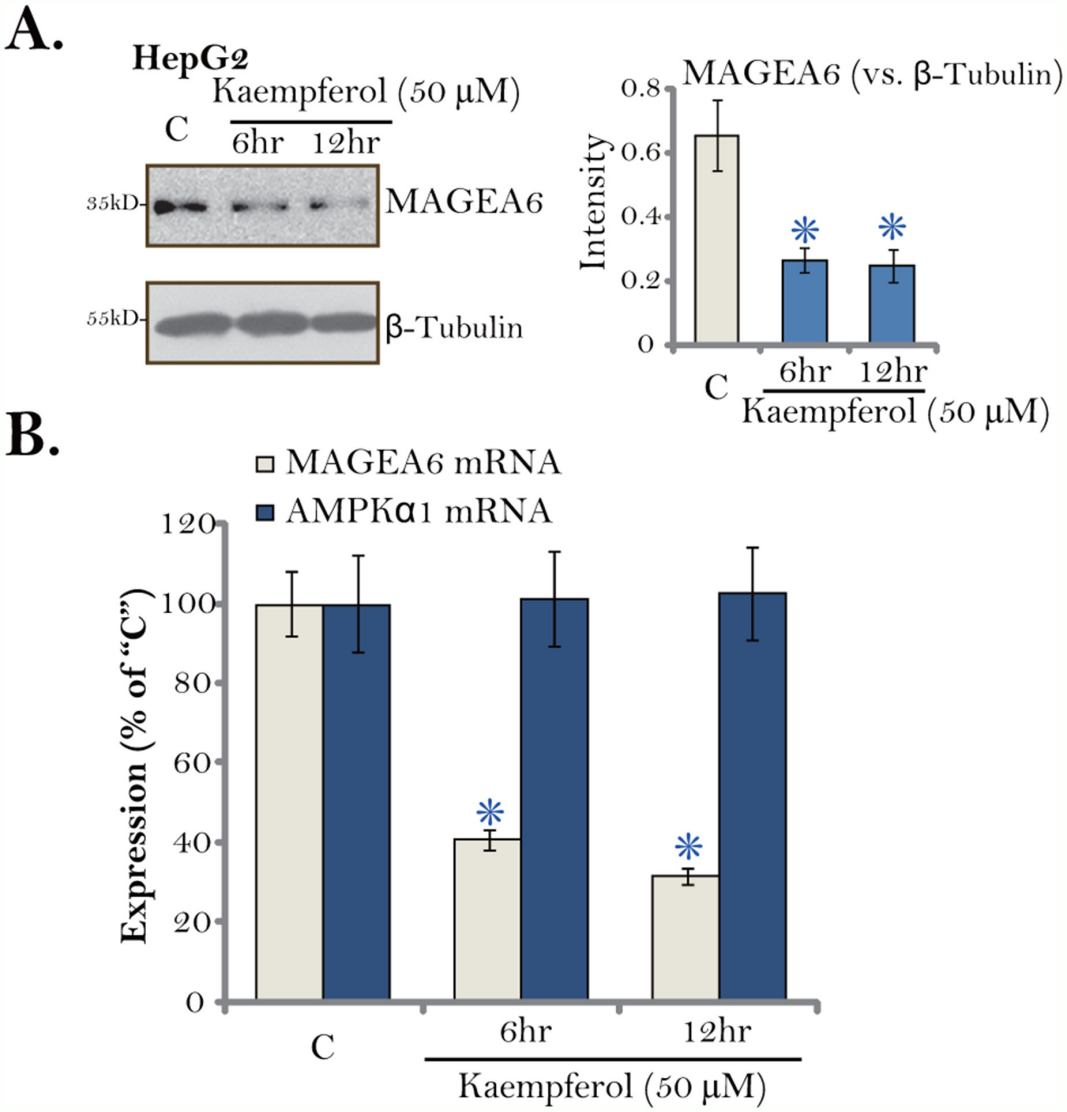
Kaempferol downregulates MAGE6 in HCC cells HepG2 cells were cultured in Kaempferol (50 μM)-containing medium for indicated time. Expressions of listed proteins were shown (**A**, data were quantified). Relative *AMPKα1 mRNA* and *MAGE6 mRNA* expressions were also tested by RT-qPCR assay **(B)**. For each assay, n=5. * *p* < 0.05 vs. “C” group. Experiments in this figure were repeated five times, and similar results were obtained.

The above results have demonstrated that AMPK activation mediated Kaempferol-induced cytotoxicity in HCC cells. We next tested the possible underlying mechanisms. We have shown that total AMPKα1 level was increased in Kaempferol-treated HCC cells (Figure 5). Pineda and colleagues have recently identified MAGEA6 as a cancer-specific AMPKα1 ubiquitin ligase [43]. We thus examined MAGEA6 expression in HepG2 cells following Kaempferol treatment. As shown in Figure 6A, MAGEA6 protein level was downregulated in Kaempferol (50 μM)-treated HepG2 cells. MAGEA6 downregulation started as early as 6 hours after Kaempferol treatment (Figure 6A, quantification). Further, real-time quantitative PCR assay (RT-qPCR) results demonstrated that *MAGEA6 mRNA* expression was also decreased by Kaempferol (Figure 6B). On the other hand, *AMPKα1 mRNA* expression was unchanged (Figure 6B). Based on these results, we hypothesize that Kaempferol downregulates MAGEA6, the AMPKα1 ubiquitin ligase, to cause AMPKα1 stabilization and accumulation. This could be at least one mechanism for following AMPK activation.

## DISCUSSION

Dysregulation and hyperactivation of mTORC1 is observed in human HCC [9, 44–46], which is a key pro-cancerous cascade required for a number of oncogenic behaviors, including cell survival, growth [47–49] and apoptosis resistance as well as angiogenesis, cell migration and cancer metastasis [9, 25, 35, 50]. Inactivation of mTORC1 represents a fine strategy to inhibit HCC cells [9, 25, 35, 50]. One main downstream following AMPK activation is mTORC1 inhibition. AMPK is shown to directly phosphorylate tuberous sclerosis complex 2 (TSC2), the mTORC1 suppressor protein, causing mTORC1 inhibition [21, 51, 52]. Intriguingly, activated AMPK could also inhibit mTORC1 in TSC2-silenced or -mutated cells [53, 54]. Later on, it was discovered that AMPK-mediated mTORC1 inhibition could also be due to its direct inhibition on Raptor [55, 56], the latter is key component of mTORC1 [53, 54]. In the current study, we show that Kaempferol treatment in HCC cells significantly inhibited mTORC1 activation, or phosphorylations of mTORC1 substrates S6K1 and 4EBP1 [57, 58]. Further, activation of AMPK mediated Kaempferol-induced mTORC1 inhibition, as AMPKα1 shRNA or dominant negative mutation restored mTORC1 activation in Kaempferol-treated HCC cells.

Although minor or moderate cell autophagy could be pro-survival, recent studies have proposed that sustained and unresolved autophagy shall promote cancer cell death [59–61]. Indeed, a number of anti-cancer agents were shown to provoke cancer cell autophagic death (but not apoptosis) [59, 61, 62]. It is an alternative way to kill cancer cells when apoptosis was deficient or blocked [59–61]. AMPK activation shall trigger autophagy activation directly and/or in-directly. AMPK directly associates, phosphorylates and activates Ulk1, which triggers cell autophagy [22]. Indirectly, AMPK-mediated mTORC1 inhibition shall remove inhibition on autophagy (by mTORC1 [22, 63]).

One novel finding of this study is that Kaempferol induced AMPK-dependent autophagy activation in HCC cells. Autophagy activation was reflected by Ulk1 phosphorylation, Beclin-1/ATG-5 upregulation and p62 degradation as well as LC3B-I to LC3B-II conversion and LC3B puncta formation. Autophagy inhibition, by 3-MA or Beclin-1 shRNA, protected HCC cells from Kaempferol. Thus, Kaempferol apparently induced HCC cell autophagic cell death (but no apoptosis). Remarkably, Kaempferol-induced autophagy activation was almost abolished by AMPK shRNA or mutation. Consequently, HCC cell death by Kaempferol was also largely attenuated. Together, AMPK activation mediates Kaempferol-induced HCC cell autophagy and subsequent cell death.

The other key finding of this study is that Kaempferol treatment induced total AMPKα1 upregulation in HCC cells. The melanoma antigen (MAGE) genes encode a large family of MAGE proteins, all sharing a common homology domain [64, 65]. MAGEA6 is expressed exclusively in the male testis with unknown functions. It is however frequently re-expressed in various human cancers, which is involved in cancer cell progression [43]. A recent study Pineda *et al.*, has confirmed MAGEA6 as the cancer-specific AMPKα1 ubiquitin ligase [43]. In the current study, we show that MAGEA6 expression is also detected in HCC cells, whose expression was downregulated by Kaempferol. MAGEA6 downregulation could be the primary reason of following AMPKα1 upregulation and activation. It will be certainly interesting to further test the mechanism of MAGEA6 downregulation by Kaempferol.

## MATERIALS AND METHODS

### Reagents

Kaempferol, 3-methyaldenine (3-MA) and puromycin were purchased from Sigma-Aldrich (Sigma, St. Louis, MO). C8 ceramide was a gift from Dr. Wang [27]. The anti-MAGEA6 antibody was obtained from Abcam (Shanghai, China). All the other antibodies were provided by Cell Signaling Tech Co. (Denver MA). The reagents for cell culture were from Gibco BRL (Suzhou, China).

### Culture of established human cell lines

The established HCC cell lines, including HepG2, Huh-7, BEL7402, and SMMC, as well as the L02 hepatocyte cell line were purchased from the iBS cell bank of Fudan University (Shanghai, China). The HCC cells and L02 cells were maintained in DMEM/RPMI with 10% FBS and necessary antibiotics [9, 35].

### Primary culture of human HCC cells and human hepatocytes

Three lines of the primary human HCC cells, namely “Pri-1/-2/-3”, were provided by Dr. Sun [25]. These primary cancer cells were derived from surgery-isolated fresh HCC tissues of informed-consent primary HCC patients [25]. Cells were cultured in the medium for primary human cells. Fibroblast cultures were abandoned. The primary human hepatocytes were provided by Dr. Fan [26], and cultured as previously described [26]. All investigations were in accordance with the principles expressed in the Declaration of Helsinki.

### CCK-8 cell viability assay

Cells were plated at a density of 2 × 10^4^ cells per well onto 96-well tissue-culture plates. Cells were further cultured in Kaempferol-containing medium for applied time. Afterwards, the cell viability was measured by Cell Counting Kit-8 (CCK-8) (Dojindo, Japan) kit with manufacturer’s protocol. The OD value of the treatment group was normalized to the control group [66].

### Trypan blue staining of cell death

Trypan blue staining assay was performed to test cell death.

### Colony formation assay

HepG2 cells were initially plated at a density of 4 × 10^5^ cells per well onto six-well tissue-culture plates. Kaempferol-containing medium was renewed every 2 days for a total of 10 days. Afterwards, HepG2 colonies were stained and manually counted under the microscope.

### FACS assay

After the applied Kaempferol treatment, cells were then stained with Annexin V and propidium iodide (PI) dyes (Biyuntian, Wuxi, China). Both early apoptotic cells (Annexin V^+^/PI^−^) and late apoptotic cells (Annexin V/PI^+^) were sorted by the Beckman Coulter fluorescence-activated cell sorting (FACS) machine. Annexin V ratio was recorded. PI distribution was also analyzed to reflect cell cycle progression.

### Caspase-3 activity assay

The CaspASE Assay System Colorimetric Kit (Promega, Nanjing, China) was applied to quantify caspase-3 activity [26]. Briefly, 20 μL of cell lysis per treatment was mixed with 40 μL caspase buffer. The reaction was started by adding 2 μL DEVD-pNA (the caspase-3 substrate), and incubated at 37°C for 2 hours [26]. The absorbance of the samples was measured spectrometrically at the wavelength of 405 nm, reflecting caspase-3 activity, which was normalized to that of untreated control group.

### Enzyme-linked immunosorbent assay (ELISA) assay of cell apoptosis

The Histone-DNA ELISA Detection Kit (Roche, Shanghai, China) was applied to test apoptosis of HCC cells/hepatocytes with applied treatment [36, 66, 67]. The ELISA OD at 405 nm was recorded.

### BrdU incorporation assay of cell proliferation

The 5-bromo-2’-deoxyuridine (BrdU) ELISA assay kit (Cell Signaling Tech, Shanghai, China) was applied to quantify cell proliferation, via the recommended protocol.

### TUNEL assay of apoptosis

Positive TUNEL staining in cell nuclei is a well-established indicator of cell apoptosis. HCC cells/hepatocytes were stained with TUNEL fluorescence dye (Biyuntian, Wuxi, China). TUNEL ratio was recorded. At least 300 cells of five random views of each condition were counted.

### Western blotting assay

Following the Kaempferol treatment, cells were homogenized in the commercial-available lysis buffer (Biyuntian, Wuxi, China). The protein lysates (30-40 μg per condition) were separated by 10-12.5% SDS-page gels, and were transferred onto polyvinylidene fluoride (PVDF) membranes (Millipore, Suzhou, China). The blots were then blocked, and incubated with designated primary and secondary antibodies. ECL Supersingnal West Pico Chemiluminescent kit was utilized to visualize the targeted protein bands.

### LC3B immunochemistry staining assay

As described previously [66, 68], HepG2 cells were fixed, and were then incubated with the primary anti-LC3B antibody (GFP-conjugated, Genepharm, Shanghai, China). LC3B GFP fluorescence was visualized under the Leica microscope. The percentage of HepG2 cells with LC3B GFP puncta (vs. total cell number, Hoechst stained) was recorded [66, 68]. For each condition, at least 100 cells in each view were counted.

### shRNA

The lentiviral participles with Beclin-1 shRNA (sc-29797-V), AMPKα1-shRNA (sc-45312-V) or scramble control shRNA (sc-108065) were purchase from Santa Cruz Biotech (Shanghai, China). HCC cells were seeded onto six-well plates at 50% confluence. After 12 hours culture in serum free medium, 20 μL/mL of the lentiviral shRNA was added for additional 24 hours. Cells were then subjected to puromycin (2.5 μg/mL, Sigma) selection for another 48 hours. The Beclin-1/AMPKα1 knockdown in the stable cells was tested by Western blotting assay.

### AMPKα1 dominant negative mutation

The dominant negative AMPKα1 (dn-AMPKα1, T172A) construct was provided by Dr. Lu P. H. at Nanjing Medical University. HCC cells were seeded onto six-well plates at 50% confluence. After 12 hours culture in serum free medium, dn-AMPKα(0.10 μg/mL medium) or the empty vector (pSuper-puro-Flag) was transfected to the HCC cells by the Lipofectamine 2000 reagent. Cells were then subjected to puromycin (2.5 μg/mL, Sigma) selection for additional 48 hours. Transfection efficiency was verified via Western blotting assay of endogenous and exogenous AMPKα1 expression.

### Real-time quantitative PCR assay

The Stat60 reagents were applied to extract total cellular RNA. The protocol of real-time quantitative reverse transcriptase polymerase chain reaction (“RT-qPCR”) assay by the ABI Prism 7600 Fast Real-Time PCR system was described previously [66]. The 2^−ΔΔCt^ method was applied to calculate relative mRNA expression level [69]. Glyceraldehyde-3-phosphate dehydrogenase (GAPDH) was always tested as the internal control [66]. The *mRNA primers* for human AMPKα1 and MAGEA6 were described early [43].

### Statistical analysis

The data were presented as mean ± standard deviation (SD). Statistical differences were analyzed by one-way ANOVA with post hoc Bonferroni test (SPSS version 18.0). Values of **p**<0.05 were considered statistically different.

## CONCLUSION

We suggest that Kaempferol inhibits human HCC cells via activating AMPK signaling. Kaempferol could be further tested as a promising anti-HCC agent.
